# Dataset for Fracture and Impact Toughness of High-Entropy Alloys

**DOI:** 10.1038/s41597-022-01911-4

**Published:** 2023-01-19

**Authors:** Xuesong Fan, Shiyi Chen, Baldur Steingrimsson, Qingang Xiong, Weidong Li, Peter K. Liaw

**Affiliations:** 1 Department of Materials Science and Engineering, The University of Tennessee, Knoxville, TN 37996 USA; 2 Imagars LLC, Hillsboro, OR 97124 USA; 3 State Key Laboratory of Pulp and Paper Engineering, South China University of Technology, Guangzhou, 510640 China

**Keywords:** Mechanical properties, Metals and alloys

## Abstract

Fracture dictates the service limits of metallic structures. Damage tolerance of materials may be characterized by fracture toughness rigorously developed from fracture mechanics, or less rigorous yet more easily obtained impact toughness (or impact energy as a variant). Given the promise of high-entropy alloys (HEAs) in structural and damage-tolerance applications, we compiled a dataset of fracture toughness and impact toughness/energy from the literature till the end of the 2022 calendar year. The dataset is subdivided into three categories, i.e., fracture toughness, impact toughness, and impact energy, which contain 153, 14, and 78 distinct data records, respectively. On top of the alloy chemistry and measured fracture quantities, each data record also documents the factors influential to fracture. Examples are material-processing history, phase structures, grain sizes, uniaxial tensile properties, such as yield strength and elongation, and testing conditions. Data records with comparable conditions are graphically visualized by plots. The dataset is hosted in Materials Cloud, an open data repository.

## Abstract (summary)

**Table:** 

Measurement(s)	Fracture Toughness; Impact Toughness; Impact Energy
Technology Type(s)	Mechanical Testing System

## Background & Summary

High-entropy alloys (HEAs) are one of the hottest fields of study in materials science in the recent decade^1–3^. These alloys draw the attention of researchers in both academia and industry across the world because they revolutionize the traditional way of alloy design in multi-component alloy systems. To appreciate the revolution brought about by HEAs, one needs to first examine their definition. Though there is no a universally agreed-on definition existing, the seminal paper by Yeh, *et al*.^4^ define them as alloys containing at least 5 elements with concentrations between 5 and 35 atomic percent. It is clear from the definition that HEAs mainly disrupt multi-component alloys. In other words, low-order systems, i.e., quaternary, ternary, and binary alloys and pure metals, are not much impacted by the concept. It is straightforward to appreciate that mixing multiple elements is essential to the design of multicomponent alloys. However, the traditional and high-entropy ways of mixing are distinct. In the traditional way, there is always one component that is dominantly higher than the rest, although there may be up to 3 other components having concentrations greater than 5 atomic percent. The dominant component is termed the solvent and the remaining is the solutes. Ni-based superalloys (e.g., HAYNES^®^ 282^®^) are perfect examples of this type. In the high-entropy way of mixing, there may be 2 or more components having dominance in concentration^1,2,4,5^. For example, all the 5 elements in the Cantor alloy, i.e., CoCrFeMnNi^5^ have the equal dominance. Accordingly, the unambiguous identifications of the solvent and solutes in HEAs are often impractical.

The high-entropy way of mixing multiple components has led to several distinctive characteristics to the alloys made thereby. First, it induces high configurational entropy in alloys, according to the equation of the configurational entropy of mixing for ideal solid solutions, $\Delta {S}_{{\rm{mix}},{\rm{ideal}}}=-R{\sum }_{i=1}^{n}{c}_{i}{\rm{ln}}\left({c}_{i}\right)$, where *R* is the gas constant, *c*_*i*_ is the molar fraction of the *i*^th^ component, and *n* is the total number of the constituent elements^2,6^. Second, alloys are stabilized to simpler phase structures than the traditional way of mixing, according to the equation of the free energy of mixing, $\Delta {G}_{{\rm{mix}}}=\Delta {H}_{{\rm{mix}}}-T\Delta {S}_{{\rm{mix}}}$, where *∆H*_mix_ is the enthalpy of mixing^1,2,6^. Therefore, single-phase solid solutions are very likely to form. Third, an abundance of elements dissolved in a solid solution result in a greater degree of lattice distortion, and thus strengthening, than ever before^2^. Sluggish diffusion^7^, cocktail effect^8^, chemical short-range ordering^9^, and chemical fluctuation^10^ are other attributes differentiating HEAs from their traditional counterparts.

It is continually reported that these effects contribute, in part, to the unique deformation mechanisms mediated by dislocation dynamics^11^, nano-twining^12^, stacking faults^13^, and phase transformation^14^, as well as remarkable mechanical properties, such as strength-ductility balance, excellent fracture and fatigue resistance^15–17^ in HEAs. Among various mechanical properties, fracture toughness is one of the most fundamental yet critical properties dictating the feasibility of these alloy in engineering applications^15,18^. It is simply because fracture toughness governs damage tolerance of materials in service. The reported fracture toughness of HEAs is rather scattered. In general, face-centered-cubic (fcc) alloys possess high fracture toughness whereas body-centered-cubic alloys are low^15^. Alloys with mixed or complex phase structures could exhibit fracture toughness ranging from low to high values depending on operative deformation mechanisms^2,15^. Another quantity closely related to fracture toughness is impact toughness or impact energy^2,15^, which is an older means of characterizing a material’s resistance to impact loads using Charpy or Izod impact tests^19^.

Given the importance of the fracture toughness and impact toughness of HEAs in shaping their potential applications, we believe that it is important to make a compilation of them. Such a compilation can bring two major benefits. First, associating materials, processing histories, microstructures, and testing conditions of HEAs with their fracture toughness and impact toughness and compiling them in one dataset allows researchers to examine various patterns, gaining insights in devising new compositions or processing routes for improved fracture resistance. Second, the compiled data, especially if evolving over time, are instrumental to applying artificial intelligence (AI) and machine learning (ML) to finding HEAs of more fracture resistance. The present work is conceived in consideration of these benefits.

## Methods

Fracture toughness reflects a material’s resistance to crack propagation. It is the critical stress intensity factor of a sharp crack where the propagation of a crack suddenly becomes unstable. The value of fracture toughness is affected by the constraint conditions at the tip of a crack, namely, the thickness of the component. Thin components impose less constraints onto the crack tip and induce plane-stress conditions. On the other hand, thick components impose more constraints and cause plane-strain conditions. For a given material, as the thickness of the component increases, its fracture toughness will first rise and then decline until reaching a steady value, which will not change much with a further increase in thickness. This lowest steady value is characteristic of the material and is deemed an intrinsic material property, known as plane-strain fracture toughness, *K*_*IC*_^19^. Under the non-plane-strain condition, fracture toughness is not regarded a material property and designated *K*_*C*_.

American Society for Testing and Materials (ASTM) standardizes the measurement of *K*_*IC*_ in its standard ASTM E399^20^. The measurement starts with measuring a conditional plane-strain fracture toughness *K*_*Q*_. Then, *K*_*Q*_ is checked against several validity requirements. If all validity checks are passed, *K*_*Q*_ is a valid *K*_*IC*_. If any of the validity requirements cannot be met, *K*_*Q*_ will stay conditional. One limitation of the fracture toughness measurement with ASTM E399 is that it is based on Linear Elastic Fracture Mechanics and thus only applicable to materials that are brittle or have very limited plasticity, which can ensure a small plastic zone size and *K*-dominance at the crack tip. For ductile materials, sample sizes may need to be impractically large in order for Linear Elastic Fracture Mechanics and *K*_*IC*_ measurements to be valid. As a result, it is extremely difficult or almost unlikely to obtain valid *K*_*IC*_ measurements for ductile materials with ASTM E399. In this case, ASTM E1820 based on the *J*-integral concept is usually used^19,21^. *J*-integral calculates the strain energy release rate, i.e., energy release per unit fracture surface area during crack propagation, in a material. Like ASTM E399, ASTM E1820 first measured a conditional critical *J*-integral, *J*_*Q*_. *J*_*Q*_ is then check against a series of validity requirements. If all met, *J*_*Q*_ is deemed a valid critical fracture energy, *J*_*IC*_. *J*_*IC*_ can be used as is to characterize a material’s resistance to crack propagation. Or it can be converted to *K*_*IC*_ with^2,15,19^

1 $${K}_{IC}=\sqrt{{J}_{IC}E{\prime} },$$

where *E*′ is the effective Young’s modulus, which equals *E* in plane-stress conditions and $E/\left(1-{v}^{2}\right)$ in plane-strain conditions, where *E* and *v* are Young’s modulus and Poisson’s ratio of the material, respectively.

Prior to the appearance of fracture mechanics as a rigorous discipline, pendulum-type impact tests, e.g., Charpy and Izod impact tests, are common ways to characterize the resistance of materials to fracture by impact loads. When a pendulum is released from a given height and impact the notched sample to fracture, and eventually swing to a peak height that is lower than the release height, certain energy is absorbed by the material in the form of mechanical work and this absorbed energy equals the potential energy difference of the pendulum at the beginning and ending heights. The absorbed energy is a measure of the material’s notch toughness. Although this type of test fails to measure intrinsic material properties and the measurements can only be used for comparative or ranking purposes, they are still popular in modern uses given the ease and low cost of operations. ASTM E23^22^ and ASTM E2248^23^ standardize impact testing of standard-sized notched bars and miniaturized Charpy V-notch specimens.

The first publication on fracture toughness of HEAs appeared in 2013, which used a non-standard nanoindentation method to measure the fracture toughness of the FeCoNiCrCuTiMoAlSiB_0.5_ alloy^24^. One year later, in 2014, the measurements with ASTM-E399 were made on the AlCoCrCuFeNi alloy by Roy *et al*.^25^. This first publication on using impact testing to characterize the impact energy of HEAs is on a series of Al_x_CoCrFeNi alloy in 2016^26^.

The data on the fracture toughness and impact energy in the present dataset are sourced through the publications from the appearance of the very first publications until the end of the 2022 calendar year. Web of Science and Google Scholar were two of the main search engines used for searching. Following downloading the publications, figures therein containing fracture toughness and impact energy data were screenshotted. The data points of the screenshots were digitized with WebPlotDigitizer version 4.5^27^ and deposited in an Excel template. Alongside, the composition, processing history, microstructure (phase structure and grain size) hardness, uniaxial tensile properties (strengths and elongations), testing conditions (temperature, test type, etc.), and references are recorded, when available in the same publication or related publications by the same research group. We intentionally refrain us from extracting the data from vastly different publications to avert any misleading, as the conditions of the same material may be significantly different among publications by different research groups. Accordingly, some fields of a given material may not have valid values, and they are filled with NA.

Additionally, the raw data in the literature may be reported in varied units. Unit conversions are applied to the data extracted, so that the data in the dataset have consistent units. For impact tests, some publication report impact energies, while others normalized the energy by the fractured surface area and termed it impact toughness. To maintain consistency, we opt to separate them out into two sub-datasets, one for impact energy and the other for impact toughness.

## Data Records

The data is saved in four worksheets of a Microsoft Excel workbook. The four worksheets are “Front page”, “Fracture toughness”, “Impact toughness”, and “Impact energy”. The Excel dataset is archived in the open-access data repository, Materials Cloud (URL: https://www.materialscloud.org/), for ready access^28^. It companions our published HEA fatigue dataset^29,30^.

**Fig. 1:**
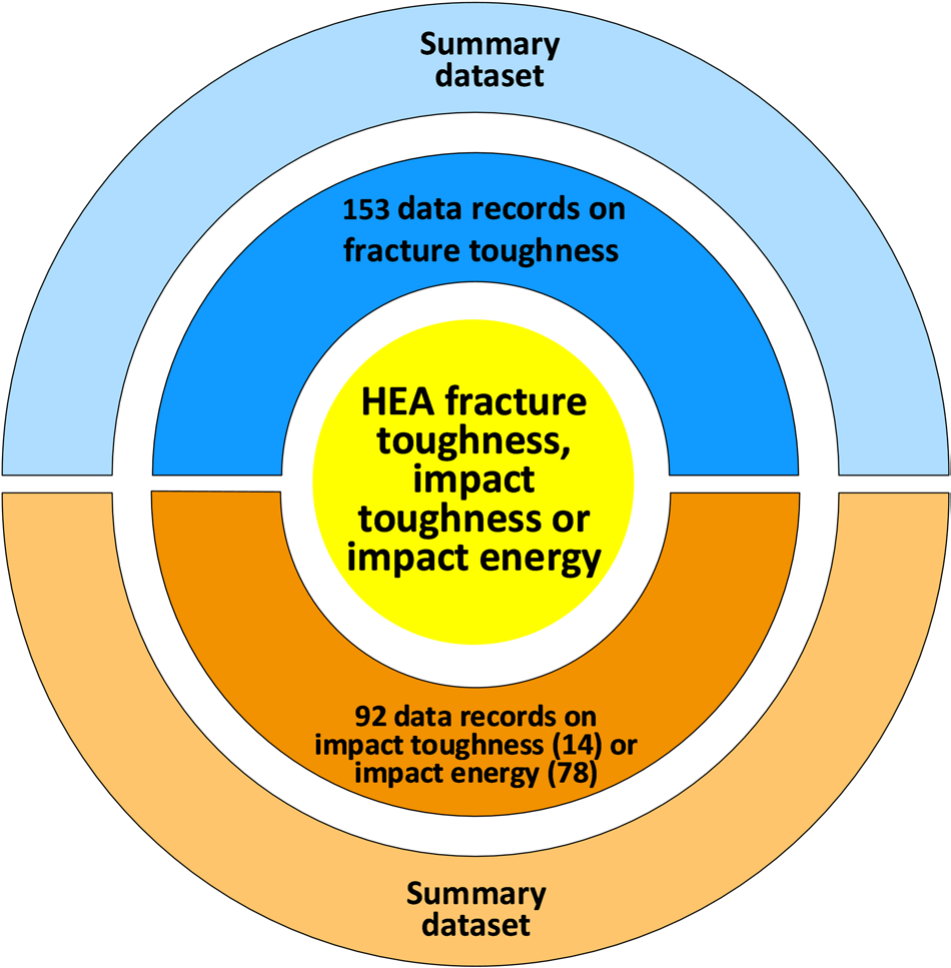
Schematic structure of the database for the fracture, impact toughness and impact energy of the high-entropy alloys, which is comprised of 153 data records for the fracture toughness, 14 data records for the impact toughness, and 78 data records on the impact energy, with each record further constituted by an extended summary covering conditions from processing all the way to testing of the fracture toughness, the impact toughness, or the impact energy.

All data in the Excel repository are subdivided into three broad categories based on the types of fracture tests conducted and the values reported, that is, fracture toughness, impact toughness, and impact energy. The three categories comprise 153, 14, and 78 data records, respectively, as schematically illustrated in Fig. 1. Each record represents a uniquely defined metallurgical condition. In other words, one composition may correspond to multiple data records, but there must exist at least one other factor (e.g., grain size) distinguishing them from each other.

**Fig. 2:**
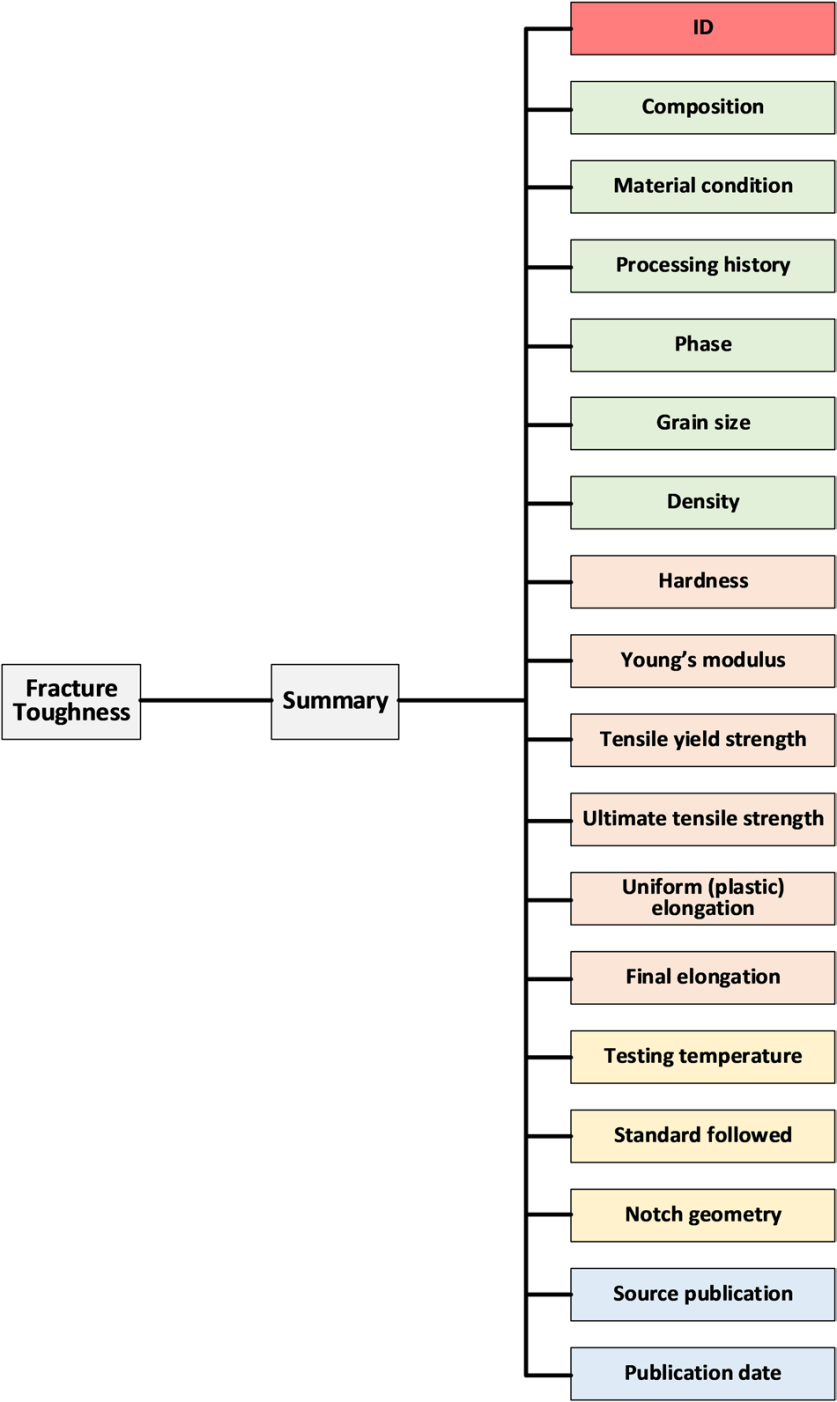
Breakdown of the data structure for the fracture toughness. Summary is categorized by color.

**Fig. 3:**
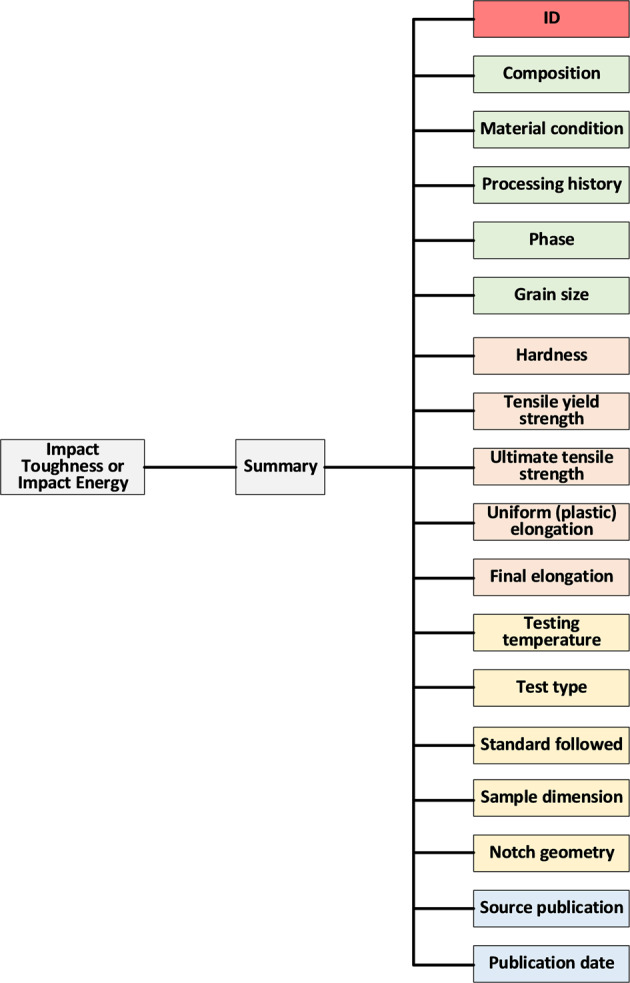
Breakdown of the data structures for the impact toughness and the impact energy.

The data structure of the fracture toughness dataset is displayed in Fig. 2. It consists of over a dozen of blocks, with each block corresponding to a column in the Excel dataset. All the data blocks may be classified to several sub-groups, i.e., alloy basic information, tensile properties, fracture-toughness testing conditions, and the source reference, as signaled by differently colored blocks in Fig. 2. Impact toughness and impact energy data have similar data structures, as illustrated in Fig. 3.

## Technical Validation

The original literature data of the same type coming in distinct units are converted to a consistent unit. The accuracy of the extracted data, derived data, and unit conversion is cross-checked and verified multiple times by the team.

Data visualization acts as an another means of data validation. All records in the individual datasets of fracture toughness, impact toughness, and impact energy are plotted to visually compare to the source plots in the literature from which the data are extracted. Any spotted discrepancies between our plots and the source ones are investigated and corrected if misrepresentation is confirmed.

**Fig. 4:**
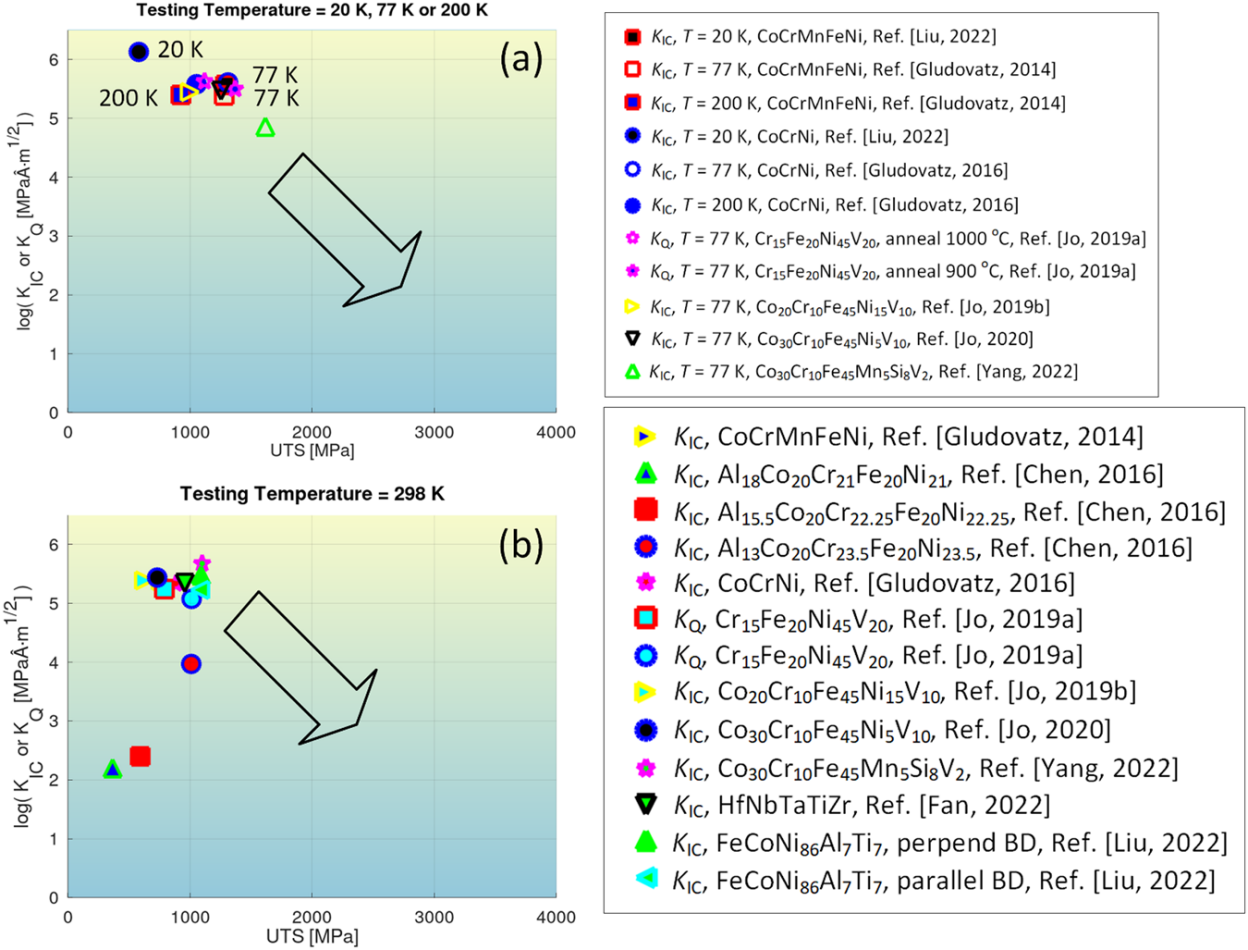
Graphical compilation of *K*_*IC*_ or *K*_*Q*_ of high-entropy alloys as a function of their ultimate tensile strengths at different testing temperatures^62–71^. (**a**) *T* = 20K, 77 K, or 200 K. (**b**) *T* = 298 K. Note that only a fraction of data in the dataset with ultimate tensile strength available is plotted. The arrows in the graph indicate a trend.

**Fig. 5:**
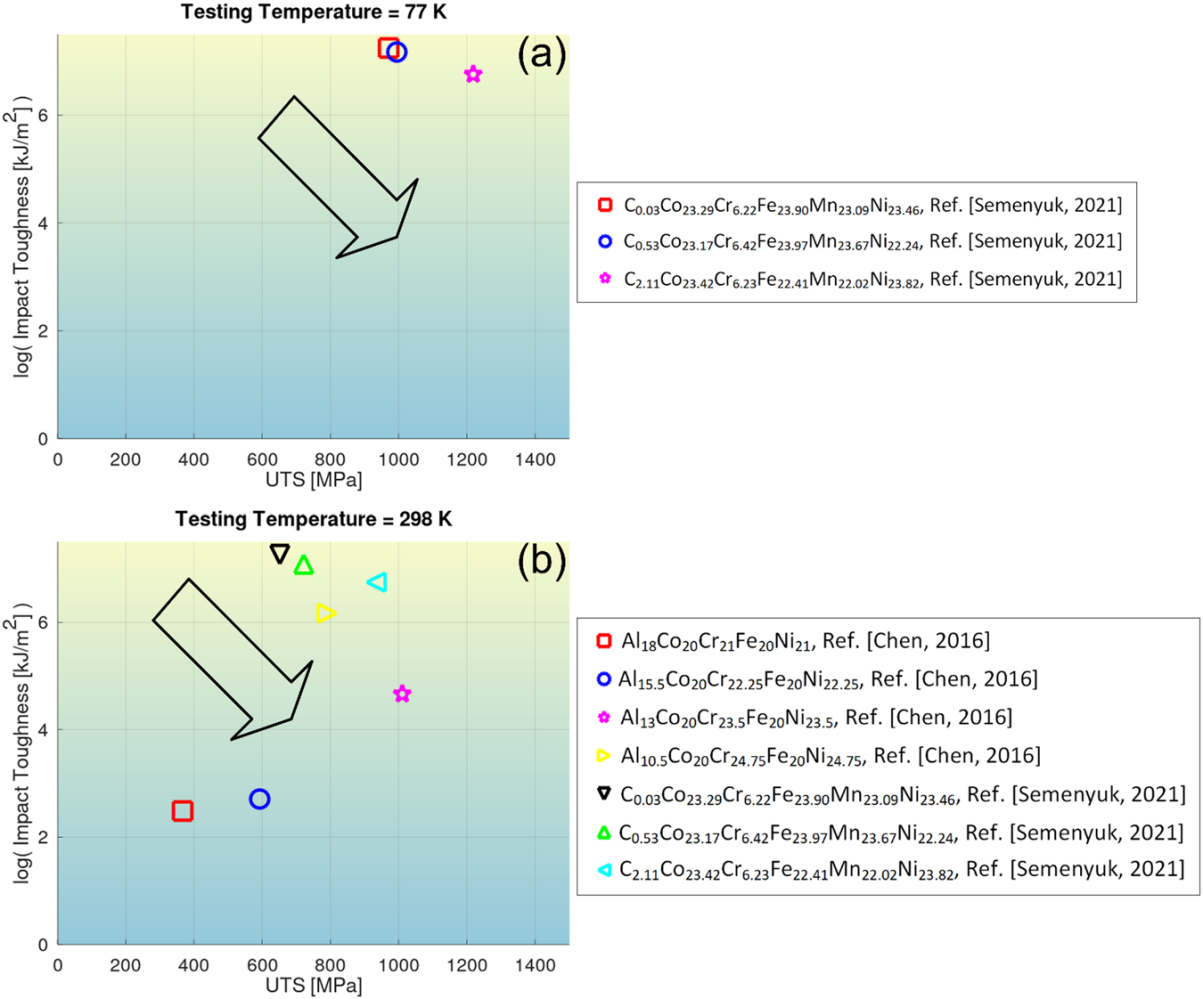
Graphical compilation of impact toughness of high-entropy alloys as a function of their ultimate tensile strengths at the testing temperatures^63,72^. (**a**) *T* = 77 K. (**b**) *T* = 298 K. Note that only a fraction of data in the dataset with ultimate tensile strength available is plotted. The arrows in the graph indicate a trend.

**Fig. 6:**
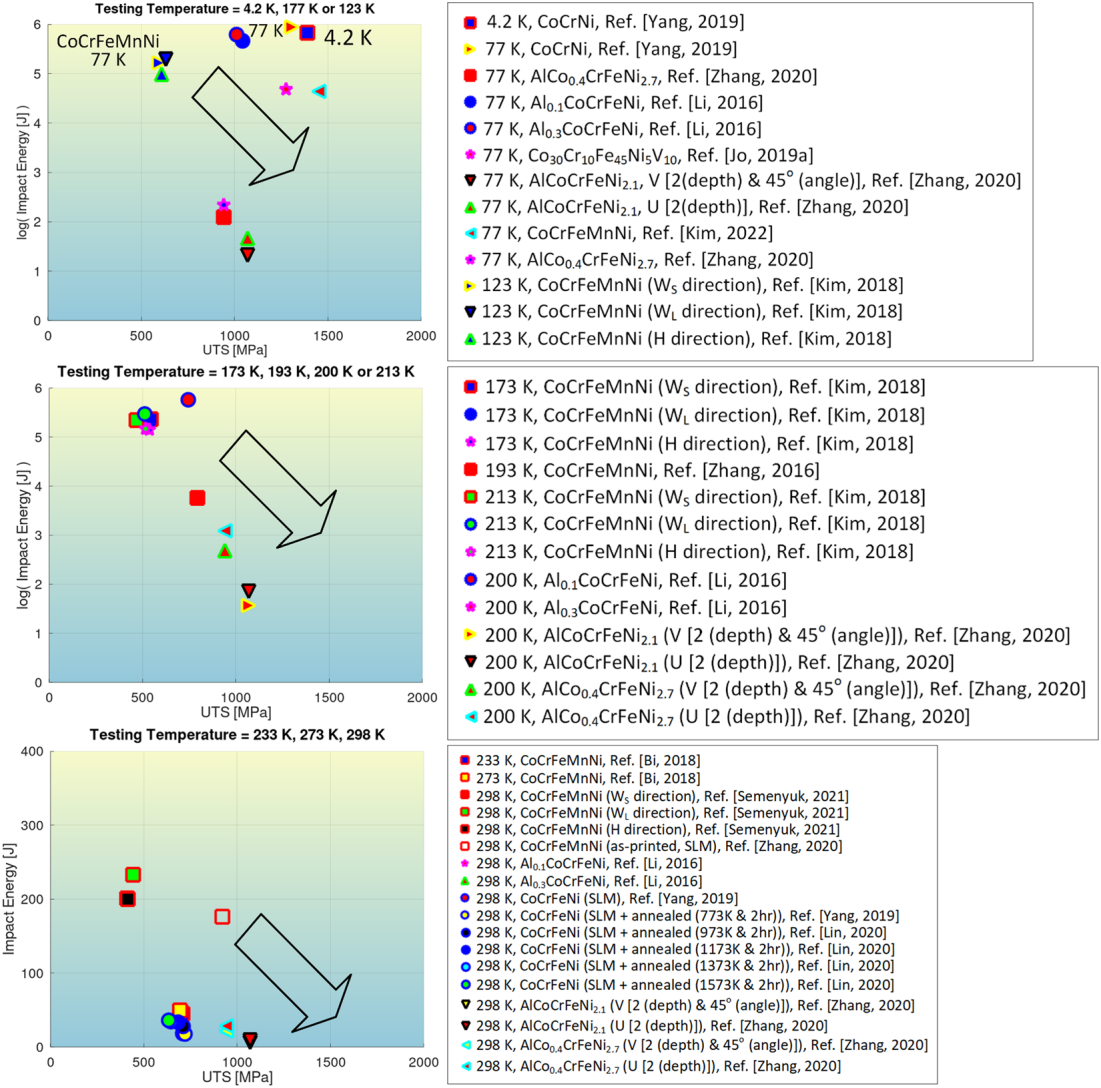
Graphical compilation of data on impact energy of high-entropy alloys at the testing temperatures of *T* = 4.2 K, *T* = 77 K, *T* = 123 K, *T* = 173 K, *T* = 193 K, *T* = 200 K, *T* = 213 K, *T* = 233 K, *T* = 273 K or *T* = 298 K^26,73–79^. Note that only a fraction of data in the dataset with the ultimate tensile strength available is plotted. The arrows in the graph indicate a trend^52,80–88^.

Some of the plots in comparable conditions are given as follows. Figure 4 depicts the correlative plot between *K*_*IC*_ or *K*_*Q*_ of high-entropy alloys and the corresponding ultimate tensile strengths (UTS) at different testing temperatures. Likewise, the selected records in the impact toughness and impact-energy datasets are visualized in Figs. 5, 6, respectively. Note that Figs. 4–6 just account for a portion of the full data in the respective dataset where UTS of the alloys are available. The records without UTS availability are unplotted. The records not graphed yet covered in the dataset^28^ are traced to refs. ^31–60,80–88^.

## Usage Notes

The data contained in the dataset may be used individually or collectively for various purposes. The basic usage may involve comparing the fracture properties of individual HEAs in the dataset with other materials of interest or with HEAs later tested. Statistical analyses may also be collectively applied to the data to identify correlations or patterns between fracture indices and materials properties, such as the phase structure and grain size. As the database continues to grow, the data may be used for AI or machine learning to, for example, facilitate the design of highly fracture-resistant alloys^61^. Furthermore, many more usage possibilities are waiting to be explored by researchers.

## Data Availability

The code for digitizing the data from the literature plots is the open-source code WebPlotDigitizer version 4.5^27^, which is freely accessible.
